# Alcoholic Hepatitis Mimicking Iron Overload Disorders With Hyperferritinemia and Severely Elevated Transferrin Saturation: A Case Report

**DOI:** 10.7759/cureus.41727

**Published:** 2023-07-11

**Authors:** Salina Munankami, Shefali Amin, Manish Shrestha, Rubina Paudel, Arpan Pokhrel

**Affiliations:** 1 General Medicine, Kathmandu Medical College, Kathmandu, NPL; 2 Internal Medicine, Reading Tower Health, Reading, USA

**Keywords:** alcohol-related liver disease, high ferritin, ferritin, cirrhosis, iron stores, severe alcoholic hepatitis

## Abstract

Iron overload disorders can present as non-specific symptoms and develop gradually but, if untreated, can be very fatal. The common causes include multiple blood transfusions for chronic anemia and increased iron absorption, including hereditary hemochromatosis (HH). HH is one of the common causes of iron overload disorders and usually presents with liver cirrhosis in a setting of significantly elevated ferritin and elevated transferrin saturation. Alcoholic hepatitis is a clinical syndrome of progressive inflammatory liver injury associated with long-term heavy intake of ethanol. However, in patients with alcohol abuse, excessive alcohol consumption can disrupt iron metabolism releasing large amounts of iron into circulation. This can cause severely elevated ferritin due to disruption of iron metabolism, simulating iron overload disorders such as HH, especially if the patient also has liver cirrhosis. Even though a high transferrin saturation of greater than 45% is recommended as a cutoff transferrin value as high sensitivity for detecting iron overload disorders, it has a low specificity and positive predictive value and often identifies people with other causes of acutely elevated ferritin levels such as alcohol liver disease and hepatitis. Recognizing this feature and timely management can spare the patient from unnecessary phlebotomies and prompt treatment for alcoholic hepatitis. We present an interesting case of severe alcoholic hepatitis mimicking HH with severely elevated ferritin levels and transferrin saturation with underlying liver cirrhosis.

## Introduction

Alcohol hepatitis is a syndrome of alcohol-associated liver disease (ALD) characterized by a systemic inflammatory response, which presents as jaundice, malaise, and tender hepatomegaly with moderately elevated transaminases secondary to heavy alcohol intake [1]. The diagnosis is based on clinical and laboratory features such as a long history of alcohol intake, jaundice, moderately elevated aminotransferases, an aspartate transferase (AST)-alanine transferase (ALT) ratio greater or equal to 2, elevated serum bilirubin, and elevated international normalized ratio (INR). The other common cases of acute hepatitis also need to be ruled out. Treatment includes conservative measures and abstinence from alcohol, and steroids, in particular instances [2].

Hereditary hemochromatosis (HH) is an autosomal recessive condition due to a mutation in the HFE gene leading to high levels of tissue iron deposition, which can also present as hepatomegaly, increased hepatic transaminases, and cirrhosis [3]. The diagnosis is based on detecting HFE gene mutation in patients with elevated ferritin and transferrin saturation. Treatment involves the removal of excess iron from the tissue, typically by phlebotomy [4].

In acute liver injuries such as alcoholic hepatitis, injury to the hepatocytes can release stored ferritin into the circulation, leading to severely elevated serum ferritin levels, serum iron levels, and transferrin saturation [5]. These findings can mimic iron overload disorders such as HH. Early recognition will prevent unnecessary phlebotomy and prompt the start of proper treatment. Transferrin saturation is a valuable parameter for the distinction between the presence or absence of iron overload in a setting of hyperferritinemia. However, it doesn't necessarily exclude the presence of an iron overload disorder [6], such as in this case. We now present an unusual presentation of severe alcoholic hepatitis mimicking iron overload disorders with hyperferritinemia and severely elevated transferrin saturation.

## Case presentation

A 38-year-old male with no past medical history presented with worsening yellowish discoloration of the eyes for three weeks. The patient denied abdominal pain, melena, hematemesis, distension, nausea and vomiting, diarrhea, constipation, leg swelling, weight loss, fatigue, rash, itching, joint pain, and fever. He reported drinking about 12 bottles of 22 ounces of beer weekly for the past 15 years. He denied any similar episodes in the past. He was not taking any medications. The patient denied tobacco use, IV drug use, history of blood transfusion, or unsafe sexual intercourse. The patient denied any family history of liver disease or gastrointestinal malignancy. On admission, vitals signs showed a pulse rate of 62/min (regular), non-tachypneic with a respiratory rate of 16/min, normotensive with a blood pressure of 124/78 mmHg, and afebrile with a temperature of 98.7°F. A physical exam was significant for bilateral scleral icterus. An abdominal exam revealed a palpable liver edge up to 3 cm below the right costal margin. The rest of the physical exam was unremarkable. There were no signs of peripheral stigmata of liver diseases such as palmar erythema, telangiectasia, gynecomastia, or symptoms of portal hypertension such as ascites and splenomegaly.

Laboratory analysis (Table 1) revealed elevated liver enzymes with a deranged hepatic function panel. HIV, salicylate level, acetaminophen level, antinuclear antibody, anti-smooth muscle antibody, anti-liver-kidney antibody, anti-mitochondrial antibody, and alpha-1 antitrypsin Ab were negative. Anti-hepatitis A virus IgM (anti-HAV IgM), hepatitis B surface antigen (HBsAg), anti-hepatitis B surface (anti-HBs), anti-hepatitis B core IgM (anti-HBc IgM), anti-hepatitis C virus (anti-HCV), and anti-hepatitis E virus (anti-HEV) were negative. However, the patient had significantly elevated ferritin at 2532 ng/mL, serum iron at 233 mcg/dL, iron saturation of 103%, and transferrin at 161 ug/dL. The CT scan of the abdomen and pelvis revealed decreased liver size, a nodular contour consistent with cirrhosis, and a small to moderate volume of ascites.

**Table 1:** Admission laboratory investigations IgM: immunoglobulin M, IgG: immunoglobulin G, CO_2_: carbon dioxide, INR: international normalized ratio, Ag: antigen

Laboratory Tests	Lab Values	Reference Range
Sodium (mmol/L)	136	136-145
Potassium (mmol/L)	3.5	3.5-5.1
Chloride (mmol/L)	97	98-107
CO_2_ (mmol/L)	30.5	21-31
Glucose (mg/dL)	107	70-99
Blood urea nitrogen (mg/dL)	6	7-25
Creatinine (mg/dL)	1.11	0.6-1.3
Calcium (mg/dL)	9.2	8.6-10.3
Anion gap (mmol/L)	9	5-12
Magnesium (mg/dL)	1.8	1.9-2.7
Albumin (g/dL)	3.6	3.5-5.7
Total protein (g/dL)	6.3	6.4-8.9
Lipase (IU/L)	50	11-82
Alkaline phosphatase (IU/L)	215	34-104
Aspartate aminotransferase (IU/L)	963	13-39
Alanine aminotransferase (IU/L)	584	7-52
Direct bilirubin (mg/dL)	9.5	0.0-0.2
Total bilirubin (mg/dL)	21	0.3-1.0
Ethanol (mg/dL)	<10	<10
Lactic acid (mmol/L)	3.8	0.6-1.4
White blood cell count (x10^3^/µl)	6.8	4.8-10.8
Red blood cell count (x10^6^/µl)	5.32	4.5-6.1
Hemoglobin (g/dL)	17	14-17.5
Hematocrit (%)	48	39-53
Platelet count (x10^3^/µl)	128	130-400
INR	2.2	0.9-1.1
Iron (mcg/dL)	233	50-212
Transferrin (mg/dL)	161	203-362
Iron saturation (%)	103	20-50
Transferrin iron binding capacity (ug/dL)	225.4	284-507
Ferritin (ng/mL)	2532	24-336
Hepatitis A IgM	Non-reactive	Non-reactive
Hepatitis A IgG	Non-reactive	Non-reactive
Hepatitis B core IgM antibody	Non-reactive	Non-reactive
Hepatitis B core total antibody	Non-reactive	Non-reactive
Hepatitis B surface Ag	Non-reactive	Non-reactive
Hepatitis C antibody	Non-reactive	Non-reactive
Hepatitis E IgM	Non-reactive	Non-reactive
Anti-nuclear antibody	Negative	Negative
Ceruloplasmin (mg/dL)	24	18-36
Anti-smooth (actin) antibody (U)	<20	<20

**Figure 1:**
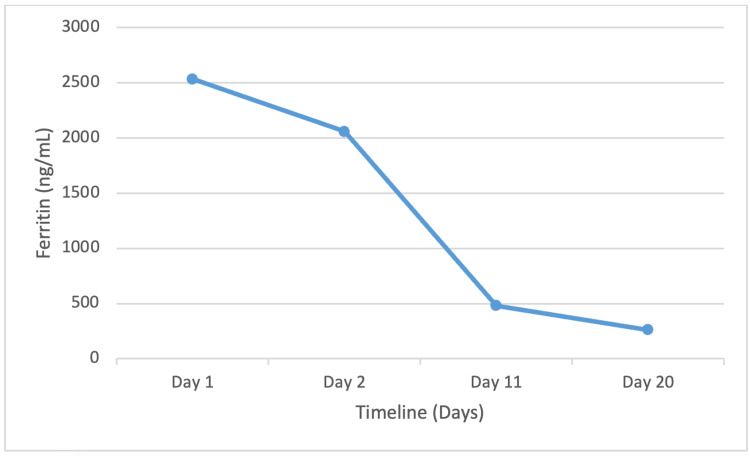
Ferritin trend

With severely elevated serum ferritin and transferrin saturation combined with imaging findings suggestive of cirrhosis, a presumptive diagnosis of hemochromatosis was made, and the patient was planned for therapeutic phlebotomy. However, the HFE genotype for HH was negative for the C282Y and H63D mutations. Following this, the patient was treated conservatively with oral prednisone for severe alcoholic hepatitis as the Maddrey discriminant function was greater than 32. On day 4, Lille score was calculated, which was more significant than 0.45, suggesting an appropriate response to steroids. The patient’s liver function improved significantly within a week, and their ferritin level normalized within three weeks (Figure 1). The patient was discharged with plans for outpatient follow-up.

## Discussion

Alcohol consumption has a wide range of manifestations, such as alcoholic fatty liver disease, alcoholic hepatitis, and cirrhosis. In addition, excessive alcohol consumption can present as acute onset of symptomatic hepatitis. Even though the amount of alcohol intake that can cause alcoholic hepatitis is unknown, most of the patients have had a history of heavy alcohol use for more than two decades [7]. Patients usually present with jaundice, anorexia, fever, and enlarged liver. Patients might also present with features of decompensated cirrhosis, such as ascites and hepatic encephalopathy, if they have underlying alcoholic cirrhosis. Patients often have tender hepatomegaly. There can be features such as palmar erythema, gynecomastia, and spider angiomata suggestive of advanced liver disease with underlying cirrhosis [8]. Laboratory analysis shows moderate elevations of aminotransferases (less than 300 IU/L) with a ratio of AST/ALT greater than 2, elevated serum bilirubin, leukocytosis with a predominance of neutrophils, and elevated INR levels [9]. Ferritin levels are usually not checked routinely in alcoholic hepatitis unless there is a suspicion of iron deficiency anemia with decreased ferritin levels or HH. However, severely elevated ferritin levels can be seen due to the release of damaged hepatocytes from liver inflammation [10]. The treatment of alcoholic hepatitis includes conservative management, alcohol abstinence, and steroids, in particular cases [7].

HH is an autosomal recessive inherited disorder caused by a mutation in the HFE gene, causing excessive tissue iron deposition. Patients typically present with nonspecific symptoms such as fatigue, hepatomegaly, and liver function abnormalities. They can also present with manifestations of organ iron overload involving the heart and endocrine organs [11]. As both HH and alcohol abuse can cause liver cirrhosis, they can have similar clinical presentations with severely elevated ferritin levels and liver dysfunction [2,4]. Even though a high transferrin saturation of greater than 45% is recommended as a cutoff transferrin value as high sensitivity for detecting iron overload disorders, it has a low specificity and positive predictive value. It often identifies people with other causes of acutely elevated ferritin levels, such as alcoholic liver disease and hepatitis [10]. Thus, ALD should be in the differential diagnosis in patients with clinical and laboratory findings suggesting iron overload disorders, such as HH with liver cirrhosis [8,12].

Serum ferritin levels usually reflect the iron status in the body in the absence of systemic inflammation [6]. Elevated serum ferritin levels indicate increased iron stores but are more commonly seen in acute phase reactions [13]. All forms of inflammation, regardless of the cause, may cause elevated ferritin levels due to stimulation of synthesis of ferritin and hepcidin from stimulation of pro-inflammatory cytokines. Clinical interpretation of elevated ferritin levels is often complex and regarded as a nonspecific marker of a wide range of pathological processes. The various etiology of hyperferritinemia are listed in Table 2. The differentiation of hyperferritinemia is essential, as the management, treatment, and prognosis greatly vary between the different entities [10].

**Table 2:** Underlying causes of hyperferritinemia with or without an associated iron overload COVID-19: coronavirus disease 2019, HHCS: hereditary hyperferritinemia cataract syndrome, HLH: hemophagocytic lymphohistiocytosis, RBC: red blood cell, HH: hereditary hemochromatosis

Hyperferritinemia without iron overload	Hyperferritinemia with or without iron overload	Hyperferritinemia with iron overload
Hepatocellular damage	Cirrhosis	HFE hemochromatosis
Metabolic syndrome	Alcoholic liver disease	Dysmetabolic iron overloading syndrome
Insulin resistance/type II diabetes mellitus	Non-alcoholic fatty liver disease	Iron-loading anemias (congenital or acquired)
Excessive alcohol consumption	Viral hepatitis	Iatrogenic iron overload (RBC transfusion, parenteral iron administration)
Inflammatory and infectious conditions (septic shock, COVID-19)	Porphyria cutanea tarda	African iron overload
Malignancy		Non-HFE HH
Benign hyperferritinemia/HHCS		Ferroportin disease
Immune-mediated syndromes (primary and secondary HLH, adult-onset Still’s disease)		Aceruloplasminemia/hypoceruloplasminemia
Gaucher disease		Atransferrinemia/hypotransferrinemia

## Conclusions

Elevated ferritin levels are a non-specific finding which is often seen in acute or chronic inflammation, alcoholic liver disease, or iron overload disorders. Substantial alcohol consumption can cause a significant elevation of ferritin levels via directly damaging hepatocytes, which can mimic iron overload disorders such as HH, especially in patients with high transferrin saturation and concurrent liver cirrhosis. Thus, early recognition of these features will prevent patients from undergoing unnecessary phlebotomy, and these patients can be promptly started with appropriate treatment avoiding further complications.
